# Development and validation of machine learning-based MRI radiomics models for preoperative lymph node staging in T3 rectal cancer

**DOI:** 10.3389/fonc.2025.1610892

**Published:** 2025-09-08

**Authors:** Xuelei Qubie, Weijuan Chen, Jun Chen, Jiangqin Ma, Xin Wei, Xiling Gu, Wei Zhang, Xiaojing He

**Affiliations:** ^1^ Department of Radiology, The Second Affiliated Hospital of Chongqing Medical University, Chongqing, China; ^2^ Department of Radiology, Sichuan Provincial Corps Hospital, Chinese People’s Armed Police Forces, Leshan, China; ^3^ Optical Engineering, Beijing Institute of Technology, Beijing, China; ^4^ Department of Pathology, The Second Affiliated Hospital of Chongqing Medical University, Chongqing, China

**Keywords:** lymph node staging, machine learning, MRI, radiomics, rectal cancer

## Abstract

**Objective:**

The present research aimed to evaluate the diagnostic performance of a magnetic resonance imaging (MRI)-based radiomics model for predicting lymph node staging in patients with stage T3 rectal cancer (RC).

**Methods:**

This retrospective study included 225 patients with RC who underwent surgical resection without neoadjuvant therapy treatment. Radiomics features were extracted from high-resolution T2-weighted imaging (T2WI) of primary tumor. Feature selection was performed using the least absolute shrinkage and selection operator (LASSO) algorithm. Five machine learning classifiers were employed to construct radiomics signatures differentiating between N0/N1 (low nodal burden) and N2 (high nodal burden) stages prediction in the training cohort. The predictive performance of each classifier was evaluated using receiver operating characteristic curve analysis, with area under the curve (AUC) comparisons conducted via DeLong’s test. Decision curve analysis (DCA) and calibration curves were utilized to assess the clinical utility and calibration performance of the developed models, respectively.

**Results:**

A total of 1,746 radiomics features were extracted from the imaging data, of which 16 features were selected to construct a radiomics signature for lymph node staging in RC. The logistic regression classifier demonstrated the best predictive performance, achieving an AUC of 0.900 [95% confidence interval (CI), 0.848–0.952] in the training cohort. The model’s robustness was further validated in the test cohort, with an AUC of 0.876 (95% CI, 0.765–0.986). DCA confirmed the clinical utility of the model.

**Conclusions:**

The radiomics model based on high-resolution T2WI provided an effective and noninvasive approach for preoperatively differentiating between N0/1 and N2 stages in stage T3 RC.

## 1 Introduction

Rectal cancer (RC) is one of the most prevalent malignant tumors of the digestive tract. It ranks as the third leading cause of cancer-related mortality worldwide (1). A substantial proportion of patients present with locally advanced RC (LARC) at initial diagnosis (2). According to the National Comprehensive Cancer Network guidelines (3), the standard treatment paradigm for LARC includes neoadjuvant chemoradiotherapy followed by total mesorectal excision (TME) after a 5–12-week interval, with optional postoperative adjuvant chemotherapy. However, a recent multicenter randomized trial demonstrated that a neoadjuvant chemotherapy-only regimen in patients with LARC at a relatively low risk of recurrence (T2N1/2, T3N0/N1) is not inferior to preoperative chemoradiotherapy in terms of disease-free survival and local recurrence rates (4). This finding underscores the clinical imperative of accurately identifying T3N0/N1 RC preoperatively, as it enables tailored, risk-adapted treatment strategies and potentially mitigates unnecessary treatment-related toxicity.

Magnetic resonance imaging (MRI) serves as the preferred method for RC staging, achieving exceptional T-stage classification accuracy of 88–99% through high-resolution soft tissue characterization (5). Nevertheless, its diagnostic performance in classifying lymph node metastasis (LNM) remains suboptimal (accuracy: <80%) and is constrained by its reliance on size-morphology criteria with inherent limitations (6). Pathological analyses revealed that 28% of metastatic nodes measure ≤3 mm in short-axis diameter (7), fundamentally challenging conventional size thresholds. While functional MRI techniques, particularly diffusion-weighted imaging (DWI), show potential for improved nodal characterization through cellularity assessment via apparent diffusion coefficient (ADC) mapping (8), their diagnostic performance remains moderate (accuracy: 66%, sensitivity: 53%, and specificity: 82%), Significant ADC value overlap between reactive and malignant nodes necessitates complementary diagnostic strategies (9).

Radiomics enables the extraction of high-dimensional quantitative features from medical images. It has emerged as a powerful tool for overcoming conventional imaging limitations (10) and may serve as a valuable adjunct in assessing LNM in RC (11). However, most existing studies have primarily focused on presence vs. absence of LNM (12), with limited exploration of predictive models in terms of specific lymph node staging (e.g., N0/N1 vs. N2). The present study aimed to develop and validate a radiomics model based on high-resolution T2-weighted MRI to differentiate between N0/N1 and N2 stages in stage T3 RC patients using multiple machine learning algorithms. It also sought to establish the clinical utility of radiomics-based nodal staging as a noninvasive diagnostic tool by systematically evaluating the predictive performance of various models. A more precise lymph node staging framework may enable risk-adapted treatment strategies, such as chemotherapy-based regimens for low-risk patients (e.g., T3N0/N1), reduce unnecessary radiotherapy exposure, minimize the risk of overtreatment, and ultimately improve patient outcomes and quality of life.

## 2 Materials and methods

### 2.1 Patients

**Figure 1:**
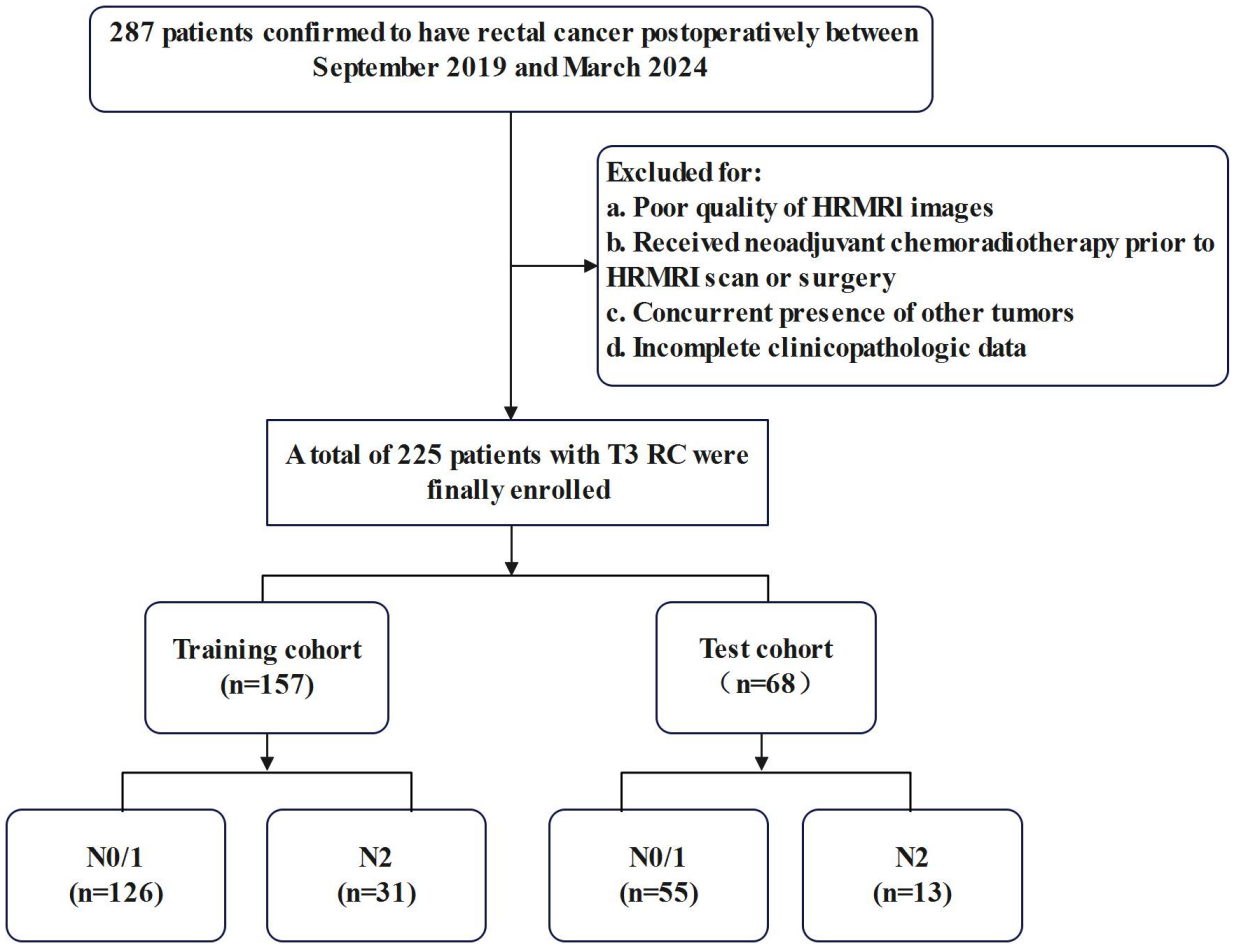
Flow chart of inclusion and exclusion criteria.

The present study initially enrolled 287 patients who underwent radical RC resection between September 2019 and March 2024. A retrospective analysis of their preoperative clinical and imaging data was carried out. The inclusion criteria were as follows: (1) postoperative pathology confirmed stage T3 RC and (2) completion of pelvic MRI within 2 weeks preceding surgery with confirmed negative circumferential resection margin. Exclusion criteria comprised: (1) incomplete MRI sequences or suboptimal image quality, (2) concurrent presence of other malignancies, (3) neoadjuvant chemoradiotherapy or other preoperative treatment regimens, and (4) incomplete clinicopathologic data. A total of 225 eligible patients were included in the final analysis after completing the screening process. The study population was randomly stratified into training (n = 157) and testing (n = 68) cohorts at a 7:3 ratio, with the detailed screening process illustrated in **Figure 1**.

The retrospective analysis was approved by the ethics committee of the Second Affiliated Hospital of Chongqing Medical University(Approval No.: 2024-79). The requirement for informed consent was waived.

### 2.2 Clinicopathological characteristics

Patient clinical characteristics were extracted from electronic medical records and comprised demographic data, such as age and sex. Tumor marker levels, including those of carcinoembryonic antigen (CEA) and carbohydrate antigen 19-9 (CA19-9), were also recorded. The normal reference ranges were defined as 0–5 ng/mL for CEA and 0–37 U/mL for CA19-9 (13).

All patients in the study underwent TME. Histopathological evaluation of stage T3 tissue specimens was performed by a pathologist with 15 years of experience. Tumor staging was conducted according to the Tumor-Node-Metastasis classification system outlined in the eighth edition of the American Joint Committee on Cancer Staging Manual (14). Specifically, LNM was categorized as follows: N0 indicated no regional LNM, N1 denoted metastasis in 1–3 regional lymph nodes, and N2 represented metastasis in four or more regional lymph nodes. Patients were stratified into the following two groups based on pathological criteria: N0–1 (low nodal burden) and N2 (high nodal burden) stages, reflecting distinct histological grades.

### 2.3 MRI data acquisition

All patients underwent rectal MRI examinations using a 3.0-T scanner (Magnetom Prisma, Siemens Healthineers, Erlangen, Germany) equipped with an 18-channel surface phased array coil. The patients fasted for 4 h prior to the examination and performed bowel preparation with a glycerol enema (20 mL). The standard rectal MRI protocols, including sagittal T2-weighted imaging (T2WI), oblique axial T2WI, coronal T2WI, and DWI with two b-factor (0 and 1,000 s/mm^2^) sequences, were conducted. The oblique axial T2WI sequence was determined in the sagittal position, which was perpendicular to the long axis of the rectal tumor according to the following parameters: field of view of 250 mm×250 mm, repetition time of 1,700 ms, echo time of 92 ms, slice thickness of 1.2 mm, flip angle of 90°, and acquisition matrix of 320×320.

### 2.4 Image evaluation

To explore the radiologic markers, two radiologists with 15 and 20 years of experience in abdominal imaging diagnosis evaluated the circumferential resection margin (CRM), extramural vascular invasion (EMVI), and distance from the anal verge. Specifically, CRM was considered to be the distance between the tumor, lymph nodes, or other lesions and the mesorectal fascia ≤1 mm (15). EMVI status was assessed using the 0–4-point grading system proposed by Jhaveri et al. (16). Patients with scores of 0–2 were classified as EMVI-negative, while others were EMVI-positive. Tumor location was measured on the approximate luminal center of the rectum on the sagittal T2WI sequence and categorized as low (0–5 cm), middle (5.1–10 cm), or high (10.1–15 cm) according to the distance from the anal verge to the lowest edge of the tumor (17).

### 2.5 Tumor segmentation

The MRI datasets were anonymized and transferred from the Picture Archiving and Communication System to a dedicated offline workstation for segmentation and subsequent analysis. Regions of interest (ROIs) were manually delineated using ITK-SNAP software (version 4.1; http://www.itksnap.org). A radiologist with 15 years of experience in abdominal imaging diagnosis outlined an ROI along the periphery of the primary rectal tumor on sequential images in oblique axial high-resolution T2WI, excluding obvious necrosis, gas, and lumen content areas. The corresponding volumetric regions of interest (VOIs) were subsequently automatically generated. The segmented VOIs were then reviewed and modified by another radiologist with 20 years of experience in abdominal imaging diagnosis in order to ensure accuracy. The radiologists were unaware of both the clinical outcomes and histopathological results. Any discrepancies in their interpretations were resolved through collaborative discussion.

### 2.6 Radiomics feature extraction

PyRadiomics software (https://github.com/Radiomics/pyradiomics) was utilized to extract a total of 1,746 radiomics features from MRI results. The radiomics features were divided into seven groups as follows: shape, first-order, gray-level co-occurrence matrix (GLCM), gray-level dependence matrix (GLDM), gray-level run length matrix (GLRLM), gray-level size zone matrix (GLSZM), and neighborhood gray-tone difference matrix (NGTDM). These quantitative radiomics features were extracted from the original, Laplacian of Gaussian (LoG), and wavelet images, which were obtained from eight decompositions after wavelet filtering. High (H)- or low (L)-pass filter application in three dimensions resulted in eight combinations as follows: LHL, HHL, HLL, HHH, HLH, LHH, LLH, and LLL. LoG images were generated by a LoG filter with a sequence of sigma values. Low and high sigma values emphasized fine and coarse textures in LoG images, respectively. Sigma values of 2, 3, 4, and 5 were utilized in the study.

### 2.7 Feature selection and model construction

A three-step procedure was performed for dimensionality reduction of radiomics features. First, radiomics features with a variance of >1.0 were selected. Second, analysis of variance was carried out in order to select the statistical influence feature. The radiomics features were available after applying the least absolute shrinkage and selection operation (LASSO) regression method, which was used to select the N-stage classification-related features with non-zero coefficients from the training cohort. The radiomics score (rad-score) was computed for each patient after feature selection utilizing the LASSO regression with a combination of selected features weighted by their respective coefficients. Five machine learning models [logistic regression (LR), support vector machine, (SVM), Bernoulli Naïve Bayes, ridge, and stochastic gradient descent (SGD)] were developed to fully exploit the potential of the remaining radiomics features. The grid search and five-fold cross-validation algorithm were used in the training dataset to select the optimal model hyperparameters. The model with the best cross-validation performance was used for further analysis. Both feature selection and radiomics signature development were performed in the training cohort. The performance of the obtained radiomics signature was evaluated using an inter-validation cohort, which was not employed for model development. Stratified cross-validation was implemented, employing a stratified sampling approach to preserve consistent class distribution across all data subsets.

**Figure 2:**
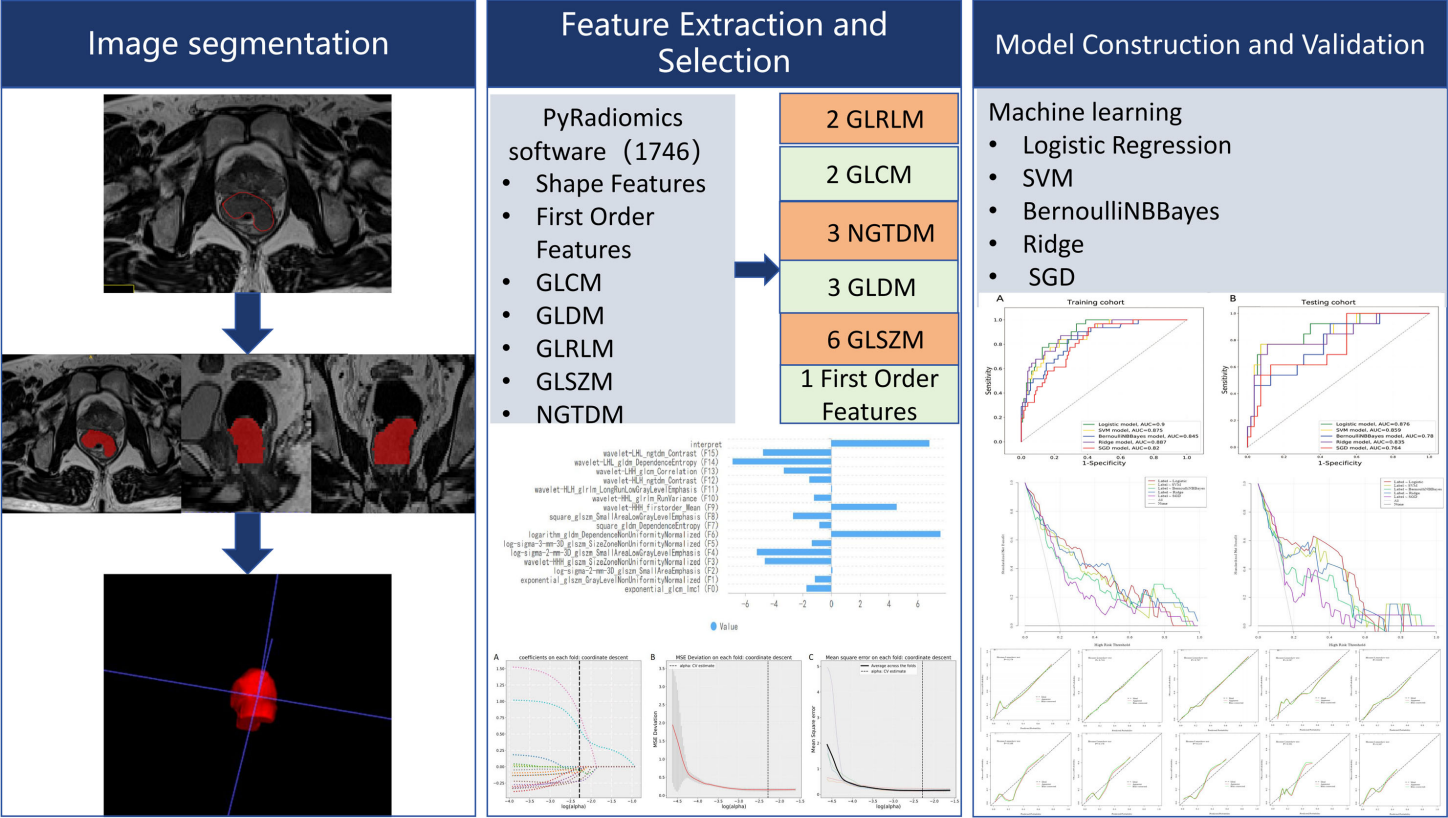
The framework for the radiomics workflow.

The processes of tumor segmentation, feature extraction, feature selection, and model validation are shown in **Figure 2**.

### 2.8 Statistical analysis

R software (version 3.5.3, http://www.R-project.org) and Python software (version 3.7.12, http://www.Python.org); were used to perform statistical analyses and model construction. Categorical variables were expressed as frequencies (percentages), and continuous variables were presented as mean ± standard deviation (SD) for normalization distribution and medians (25% quantile, 75% quantile) for other variables. Categorical variables were analyzed using a χ2 or Fisher’s exact test. The Kolmogorov-Smirnov method was used to test the normality of all measurement data. An independent sample *t*-test or Mann-Whitney U test was used to measure statistical differences. Receiver operating characteristic (ROC) curve, sensitivity, and specificity analyses were performed to compare the performance of five machine learning models. To evaluate model performance under class imbalance, the F1-score was employed. The Matthews correlation coefficient (MCC), calculated from the four categories of the confusion matrix (true positives, false positives, true negatives, false negatives), was used for binary outcome assessment. The DeLong’s test was used to compare the models’ discrimination abilities. Calibration analysis and the Hosmer-Lemeshow test were utilized to examine the agreement between the observed N stage and prediction probabilities. Decision curve analysis (DCA) was performed to determine the net benefits in clinical application of the constructed models. A p-value of < 0.05 was considered statistically significant.

## 3 Results

### 3.1 Clinical baseline characteristics

A total of 225 patients with stage T3 RC were included in the study. The cohort comprised 90 female and 135 male patients with an age range of 24–85 years. Among them, 181 cases were in N0/N1 stage, and 44 cases were in N2 stage. No statistically significant difference was found between the two groups in terms of clinical characteristics. Detailed patient characteristics and statistical results are shown in **Table 1**.

**Table 1:** Patients’ clinical characteristics.

Parameter	N0/N1(n = 181)		N2(n = 44)		Statistical values	*P*-Value
	Training cohort	Test cohort	Training cohort	Test cohort		
Age	64.00 (58.00, 71.05)	65.00 (56.20, 71.60)	59.00 (53.00, 71.60)	68.00 (65.00, 73.60)	-0.216	0.829
Sex						
Female	49(38.89 %)	29(52.73 %)	10(32.26 %)	2(15.38 %)	3.692	0.055
Male	77(61.11 %)	26(47.27 %)	21(67.74 %)	11(84.62 %)		
CEA(ng/mL)						
>5	39(30.95 %)	15(27.27 %)	10(32.26 %)	2(15.38 %)	0.112	0.738
≤5	87(69.05 %)	40(72.73 %)	21(67.74 %)	11(84.62 %)		
CA199 (U/mL)						
>37	15(11.90 %)	6(10.91 %)	6(19.35 %)	0(0.00 %)	0.139	0.710
≤37	111(88.10 %)	49(89.09 %)	25(80.65 %)	13(100.00 %)		
EMVI						
Positive	39(30.95 %)	5(9.09 %)	10(32.26 %)	6(46.15 %)	2.630	0.105
Negative	87(69.05 %)	50(90.91 %)	21(67.74 %)	7(53.85 %)		
Tumor location						
Upper	36(28.57 %)	21(38.18 %)	10(32.26 %)	1(7.69 %)	1.153	0.562
Middle	54(42.86 %)	21(38.18 %)	13(41.94 %)	9(69.23 %)		
Lower	36(28.57 %)	13(23.64 %)	8(25.81 %)	3(23.08 %)		

CEA, carcinoembryonic antigen; CA-199, carbohydrate antigen 19-9; EMVI, extramural venous invasion.

### 3.2 Models construction and validation

#### 3.2.1 Model construction

**Figure 3:**
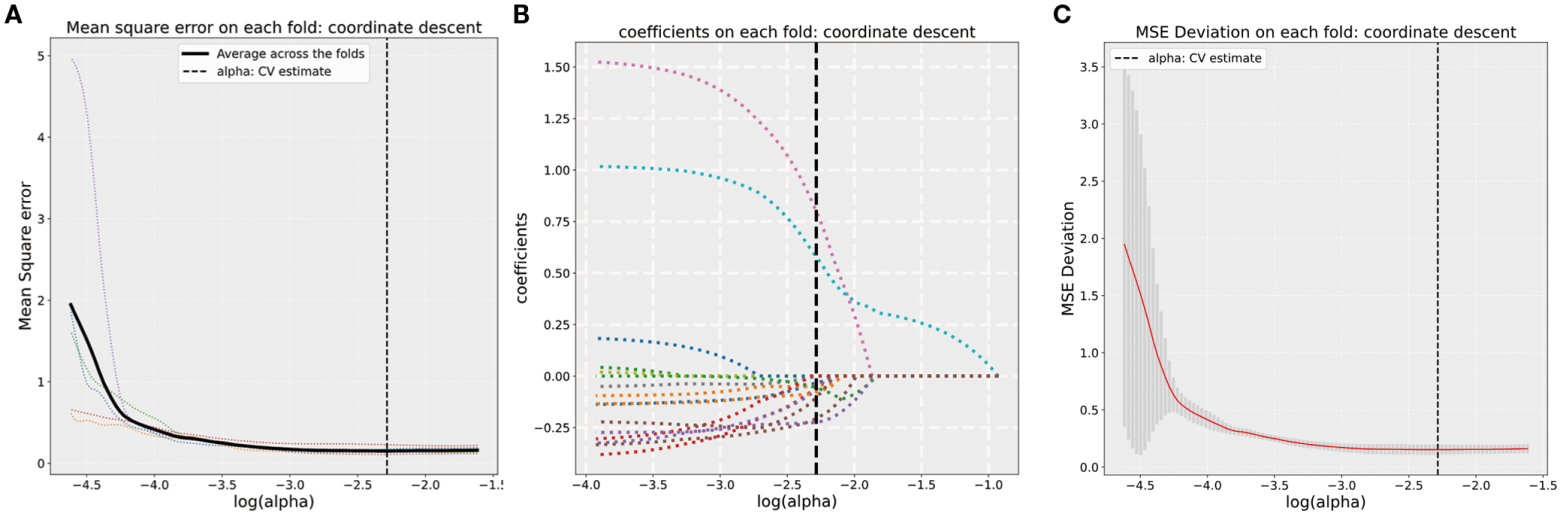
**(A)** Five-fold cross-validation results for alpha selection. The optimal alpha is marked by the dashed line. **(B)** Coefficient values corresponding to the optimal α value and selected features with non-zero coefficients. **(C)** MSE deviation on each fold using coordinate descent in five-fold cross-validation.

A total of 1,746 radiomics features were successfully extracted from T2W images for each patient. LASSO regression analysis was utilized to select radiomics features with coefficients of >0, resulting in a final retention of 16 features, as shown in **Figure 3**. The detailed feature names and their corresponding rad-score values are listed in **Supplementary Figure 1**,including 15 texture (two GLRLM, two GLCM, three NGTDM, three GLDM, and six GLSZM) and one first-order features. The features were significantly different between the N0/1 and N2 groups (all p < 0.05), except for feature F9 (wavelet-HHH_firstorder_Mean) (**Supplementary Figure 2**).

Five different radiomics signature models for predicting lymph node staging were then constructed using the above selected features based on LR, SVM, Bernoulli Naïve Bayes, ridge, and SGD classifiers in the training dataset.

#### 3.2.2 Predictive performance and validation of the model

**Figure 4:**
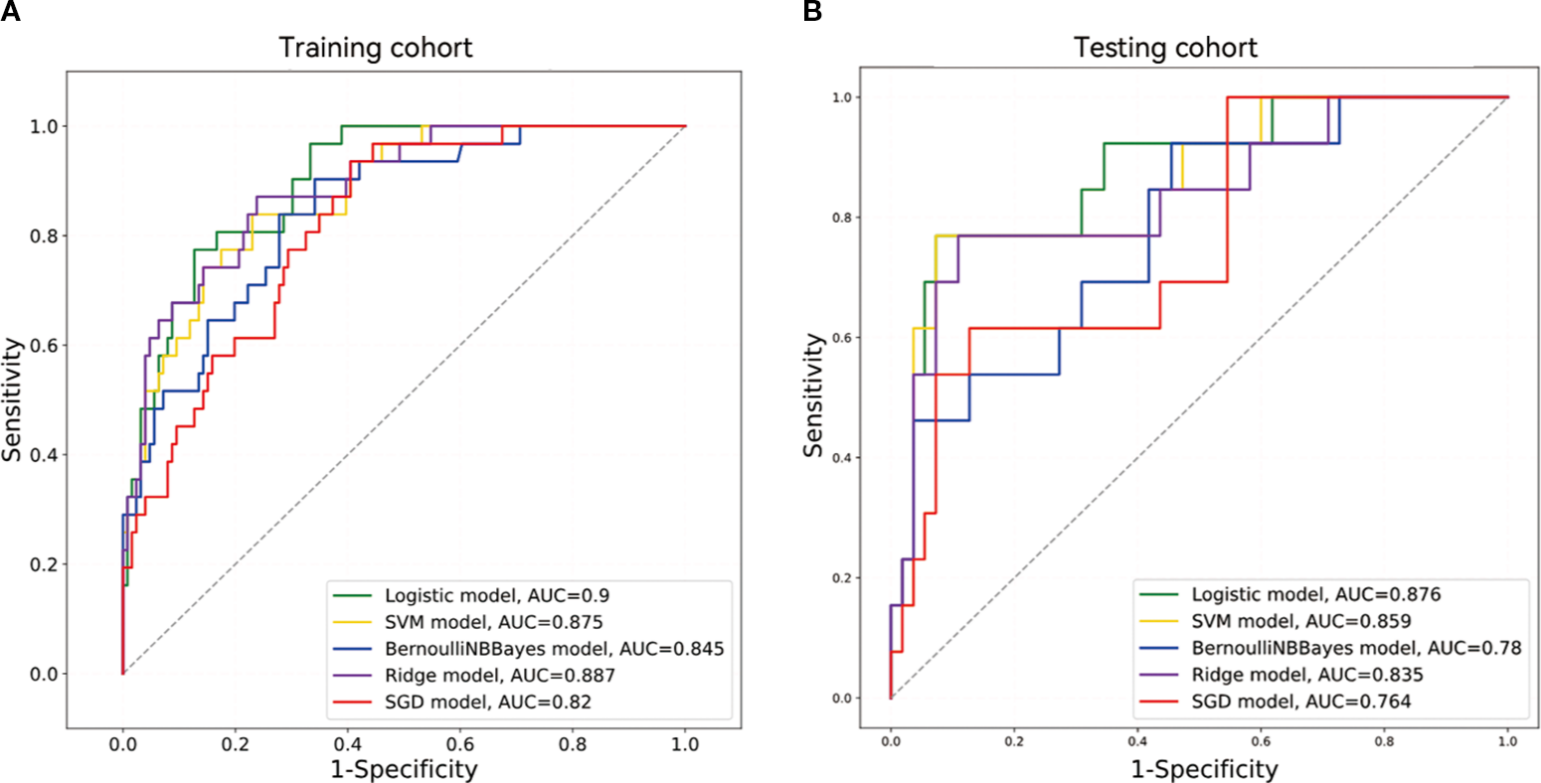
ROC curves based on five machine learning models in the training and testing cohorts.

**Table 2** summarizes the five models’ sensitivity, specificity, F1-Score, MCC, PPV, NPV, accuracy, and AUC data, with the corresponding ROC curves depicted in **Figure 4**. Among the five machine learning classifiers, the LR model performed the best in both the training and test sets, with respective AUC values of 0.900 [95% confidence interval (CI), 0.848–0.952] and 0.876 (95% CI, 0.765–0.986). The corresponding accuracy values across the two cohorts were 0.847 (95% CI, 0.843–0.852) and 0.882 (95% CI, 0.873–0.892).

**Table 2:** Radiomics signature model performance.

Model Type	AUC(95%CI)	Accuracy	Sensitivity	Specificity	F1-Score	MCC	PPV	NPV
Logistic								
Training set	0.900(0.845, 0.952)	0.847	0.742	0.873	0.657	0.567	0.590	0.932
Test set	0.876(0.765, 0.986)	0.882	0.692	0.927	0.692	0.620	0.692	0.927
SVM								
Training set	0.875(0.812, 0.939)	0.777	0.806	0.770	0.588	0.483	0.463	0.942
Test set	0.859(0.737, 0.980)	0.882	0.692	0.927	0.692	0.620	0.692	0.927
Bernoulli Naïve Bayes								
Training set	0.845(0.771, 0.918)	0.701	0.871	0.659	0.535	0.424	0.386	0.954
Test set	0.780(0.640, 0.920)	0.603	0.846	0.545	0.449	0.309	0.306	0.937
Ridge								
Training set	0.887(0.825, 0.949)	0.777	0.839	0.762	0.598	0.499	0.464	0.950
Test set	0.835(0.696, 0.974)	0.853	0.692	0.891	0.643	0.553	0.600	0.924
SGD								
Training set	0.820(0.747, 0.893)	0.656	0.903	0.595	0.509	0.397	0.354	0.962
Test set	0.764(0.618, 0.909)	0.809	0.538	0.873	0.519	0.400	0.500	0.900

F1-Score, harmonic mean of precision and recall; MCC, Matthews correlation coefficient; PPV, positive predictive value; NPV, negative predictive value.

Differences in the AUCs among the five models were compared using the DeLong’s test. The LR model significantly outperformed the Bernoulli Naïve Bayes and SGD models in the training cohort (p < 0.05). There was a significant difference in AUC values between the LR and Bernoulli Naïve Bayes models in the test cohort (p < 0.05). Further details are provided in **Supplementary Table 1**.

**Figure 5:**
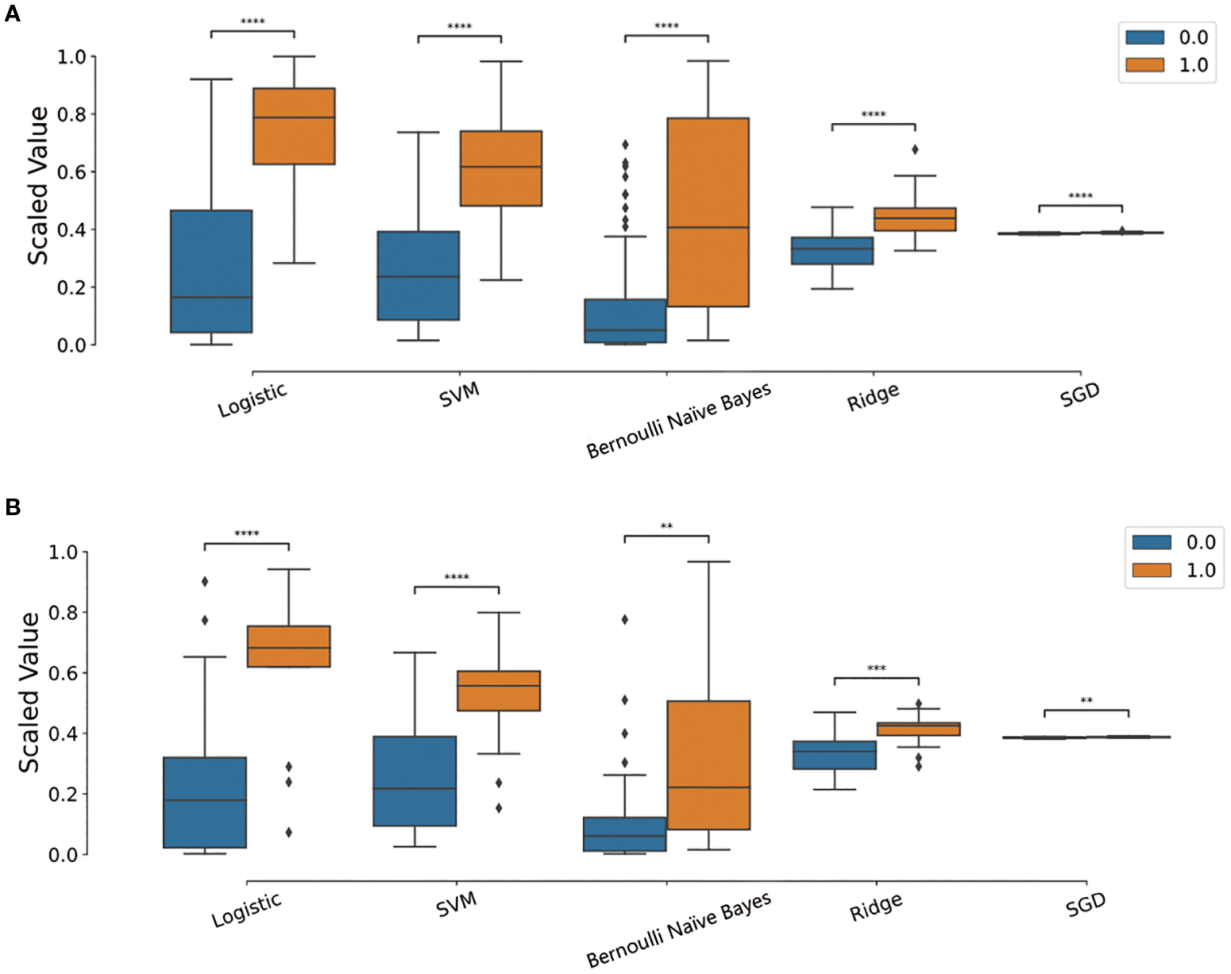
**(A, B)** Boxplots of corresponding radiomics scores in the training and testing cohorts. 0 (blue) represents N0/1 group, 1 (orange) represents N2 group. Asterisk (*) indicates level of statistical significance between categories, with more asterisks representing a higher level of significance. ("***p < 0.001, **p < 0.01, *p < 0.05").

The rad-scores in the training cohort were significantly higher in the N2 group compared to those in the N0/1 group, which was consistently validated in the test cohort, as shown in **Figure 5**. The Radscores derived from five classifier for each patient in the training and test cohort datasets are depicted in **Supplementary Figure 3**. LR and SVM demonstrated robust discriminative performance in both the training and test cohorts.

#### 3.2.3 Apparent performance and clinical use of the radiomics signature model

**Figure 6:**
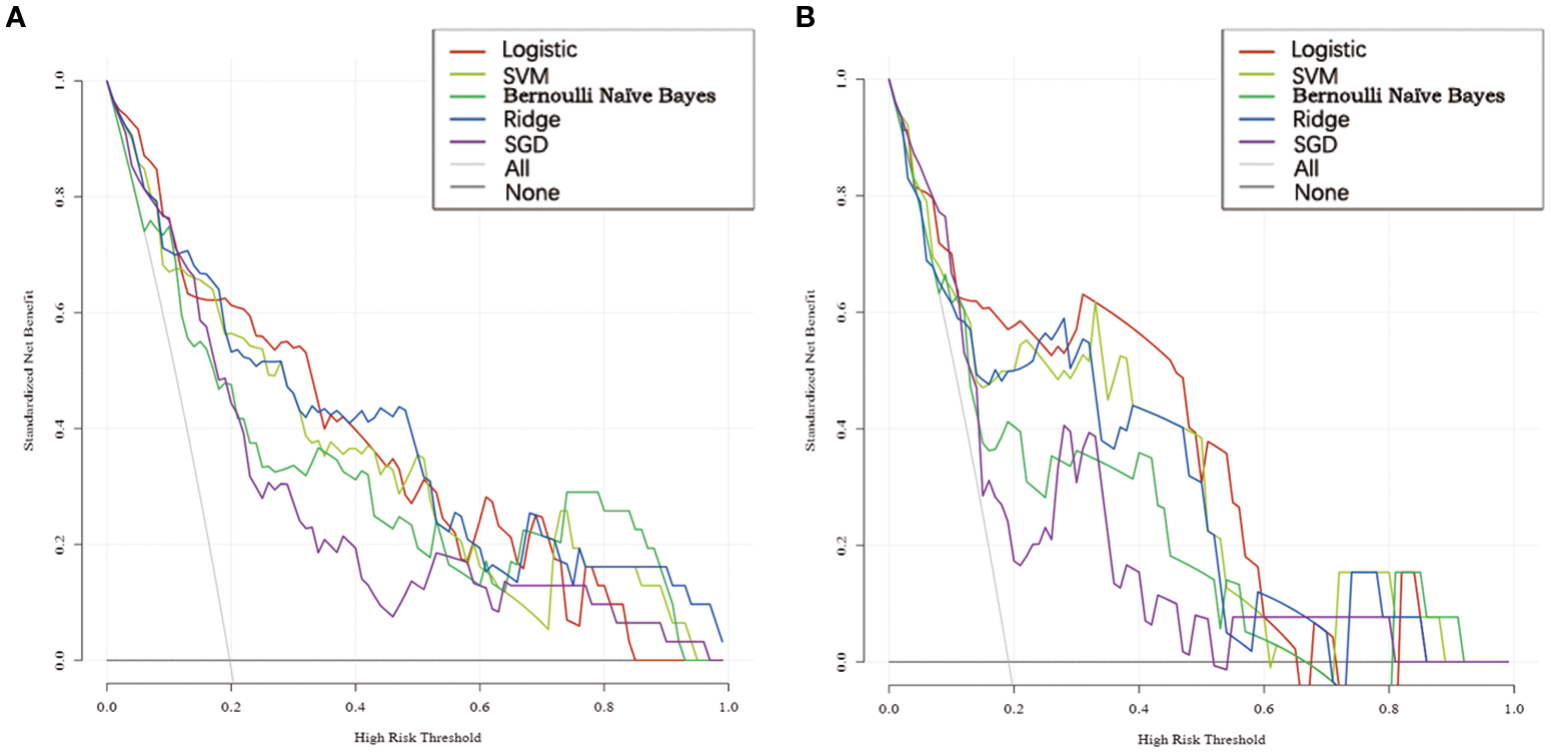
DCA for five models in the training and testing cohorts. **(A)** DCA of five models in the training cohort. **(B)** DCA of five models in the testing cohort.

The calibration curve of the radiomics models for the predicted lymph node staging in stage T3 RC showed good agreement between the observed outcomes and predicted probabilities in all datasets (**Supplementary Figure 4**). The p-values obtained from the Hosmer-Lemeshow test were all >0.05 and not statistically significant. The DCA of the radiomics signature models is presented in **Figure 6**. The DCA showed satisfactory positive benefits of the nomogram on most of the threshold probabilities, indicating a favorable potential clinical effect of the models.

#### 3.2.4 Diagnostic performance of initial imaging interpretations

Among the 225 patients evaluated, initial imaging interpretations classified 157 as N0/N1 and 68 as N2. This approach achieved a sensitivity of 54.55%, specificity of 75.69%, and an overall accuracy of 71.56%. The corresponding AUC was 0.651, indicating modest discriminative performance.

## 4 Discussion

The lymph node status in RC is a critical factor in determining the necessity of adjuvant therapy and surgical resection (18). However, Initial MRI radiology report for lymph node (LN) staging in rectal cancer relies on subjective size-morphology criteria, often showing poor interobserver agreement (κ = 0.416) and limited accuracy (AUC: 0.60–0.62), remains challenging (19). In contrast, Dong et al. (20)demonstrated that a radiomics model achieved higher diagnostic precision (PPV: 75.9%) by quantifying tumor heterogeneity, overcoming the limitations of traditional MRI. Building upon the recently published novel perspectives and conclusions (4), an exploratory investigation was conducted in the present study. Five radiomics models were developed based on high-resolution T2WI features using five different machine learning algorithms in order to noninvasively differentiate between N0/N1 and N2 stages in stage T3 RC. The performance and clinical utility of these models were systematically compared. The results showed that the LR model exhibited the best performance in predicting lymph node staging in stage T3 RC. The clinical applicability of this model was further validated via calibration curves and DCA, indicating positive clinical benefits at most threshold probabilities.

Radiomics features can reveal subtle changes in rectal tumor lesions that are difficult to discern with the naked eye (21, 22). In the present study, 1,746 radiomics features were extracted from oblique axial T2W images. LASSO regression was subsequently utilized to select 16 key features for in-depth analysis. These included one first-order and 15 texture features, with texture features being the most abundant, accounting for 93.75% of the total. This result is consistent with those described by Li and Yin et al. (23), who demonstrated that T2WI-based texture features have high accuracy in predicting LNM in RC (AUC of 0.805), further validating the importance of texture features in tumor assessment.

GLSZM features were predominant in the present study. GLSZM quantifies the size distribution of continuous image regions, finely characterizing the distribution patterns of homogeneous regions of different sizes within tumors. Smaller regions may correspond to densely packed tumor cells or micro-nodules, while larger continuous regions may reflect necrotic areas or fibrotic changes. (24). This spatial heterogeneity is closely related to tumor proliferation activity and invasiveness. Studies have shown that the short-zone emphasis in GLSZM features is significantly negatively correlated with tumor microvascular density, while large-zone emphasis is positively correlated with stromal fibrosis (25). This ability to quantify spatial heterogeneity in tumor microstructures makes GLSZM features an important indicator for assessing tumor invasiveness and metastatic potential. Therefore, texture features can capture the microscopic heterogeneity within tumors, reflecting the spatial arrangement and gray-level distribution patterns of tumor cells (26–28). These features showed significant differences in distinguishing between N0/N1 and N2 stages (p < 0.05) in the present study, indicating that LNM is closely related to microscopic structural changes within the tumor. Additionally, the predictive model based on T2WI radiomics features was constructed using wavelet features (8/16). Wavelet transform is a multi-scale analysis method with perfect reconstruction capability, ensuring no information loss or redundancy during signal decomposition. It can decompose images into high-frequency (heterogeneity) and low-frequency (homogeneity) components, facilitating the extraction of structural information and details from the original images (29). Previous studies (30) have also reported the effectiveness of wavelet features in predicting lymph node status in T2WI. Furthermore, He et al. (31) found that wavelet features in T2WI performed well in RC tumor grading, further demonstrating that they can represent the biological behavior and heterogeneity of tumors.

A growing body of evidence underscores the value of clinical parameters as complementary predictors in radiomics-based models, with integrated frameworks often demonstrating superior discrimination compared with radiomics alone (12). For example, (32) reported that a combined clinical–radiomic model improved sensitivity (82.6% vs. 78.3%) and specificity (88.9% vs. 57.9%) relative to a purely radiomic approach, while also mitigating the subjectivity of conventional MRI interpretation through visualized risk maps. Such clinical metrics may encode systemic or tumor-related biological processes that radiomics, which predominantly captures spatial and textural heterogeneity, cannot fully represent. In our cohort, however, none of the routinely collected clinical variables demonstrated statistically significant intergroup differences across outcome categories (all P > 0.05). This absence of discriminative signal suggests that, in this specific population, these variables may have limited incremental value for the prediction task. From a modeling perspective, the inclusion of non-informative covariates in high-dimensional feature spaces risks diluting true signal, inflating model variance, and impairing generalizability—particularly in datasets of modest size. Given these considerations, and in pursuit of parsimony, we elected to exclude clinical parameters from the final model. Several factors may underlie this discrepancy with prior studies. First, differences in patient demographics, disease stage distribution, and treatment patterns between our cohort and other trials could attenuate the predictive contribution of clinical variables. Second, sample size constraints may have limited statistical power to detect subtle effects, especially for variables with low intergroup variability. Third, the endpoints examined—derived from imaging-based nodal staging—may be more tightly coupled to local morphologic features than to systemic clinical markers. These hypotheses warrant systematic evaluation in larger, multi-institutional datasets, ideally with harmonized variable definitions and broader biological characterization, to clarify the true translational potential of integrating clinical and radiomic predictors.

The widespread application of machine learning algorithms in the field of radiomics has significantly improved diagnostic performance (33). Selecting the appropriate classifier is crucial for building high-performance predictive models. The present study systematically evaluated five supervised learning models commonly used for binary classification tasks, including LR, SVM, Bernoulli Naive Bayes, ridge regression, and SGD. The results showed that the LR model performed the best, with AUCs of 0.900 and 0.876 in the training and testing sets, respectively. Additionally, the model demonstrated excellent accuracy (training set: 0.847, testing set: 0.882) and specificity (training set: 0.873, testing set: 0.927). The superior performance of the LR model could be attributed to its ability to effectively handle linearly separable data, low complexity after feature selection, and resistance to overfitting. Moreover, the LR model has strong interpretability, allowing for the quantification of each feature’s contribution to the prediction results (34), which provides important references for clinical decision-making. Similarly, Wei et al. (35) developed and validated a clinical radiomics model by combining T2W and amide proton transfer-weighted MRI radiomics features, achieving efficient LNM prediction in rectal adenocarcinoma using an LR classifier (AUCs of 0.983, 0.864, and 0.851 in the training, validation, and testing sets, respectively). Cui et al. (36) used an LR model to predict a complete pathological response in LARC and achieved an AUC of 0.90. However, the LR model is not suitable for all scenarios, as classifier performance highly depends on the distribution characteristics of the training and testing sets. Qu et al. (37) found that the SVM classifier performed best when constructing a predictive model using T2W images, with AUCs of 0.892 and 0.71 in the training and validation sets, respectively. This is because SVM can handle nonlinear relationships through kernel functions and exhibits strong classification capabilities (38).

This study has several limitations. First, this study focused on evaluating MRI-based radiomics for lymph node staging in patients undergoing upfront surgical resection for stage T3 rectal adenocarcinoma. However, select patients with low-risk, distally located tumors (e.g., T3N0/N1) achieving a clinical complete response after neoadjuvant therapy may be eligible for a watch-and-wait (W&W) organ-preserving strategy. Excluding such cases may limit the model’s applicability in settings where nonoperative management is considered. Second, the model was developed solely on T2-weighted MRI data, potentially limiting sensitivity for detecting small or morphologically subtle lesions. Future work should explore the integration of functional imaging sequences—such as diffusion-weighted imaging or contrast-enhanced MRI-within a multiparametric framework to enhance lesion conspicuity and improve diagnostic accuracy. Third, manual ROI delineation inevitably introduces interobserver and intraobserver variability, which may bias feature extraction and subsequent model performance. The adoption of automated or semi-automated segmentation algorithms could reduce subjectivity, standardize feature generation, and improve reproducibility across centers. Finally, the modest sample size and absence of external validation restrict the model’s generalizability. Rigorous validation using large, multicenter datasets with diverse patient populations is essential to confirm robustness, refine calibration, and establish the clinical utility of the proposed model.

## 5 Conclusion

The present study confirmed the effectiveness of a machine learning-based high-resolution T2WI radiomics model in predicting lymph node staging in stage T3 RC. Radiomics is a noninvasive assessment method that can provide a valuable alternative for lymph node staging, supporting personalized treatment decisions. It showed broad application prospects in optimizing treatment pathways, avoiding overtreatment, and improving the prognosis of patients with LARC. Future research may further advance the clinical application of this technology via multimodal imaging and large-scale validation.

## Data Availability

The original contributions presented in the study are included in the article/**Supplementary Material**. Further inquiries can be directed to the corresponding authors.

## Supplementary material

Supplementary Figure 1 Histogram of radiomics scores based on selected features.

Supplementary Figure 2 Boxplots of 16 radiomics features in N0/1 and N2 groups.

Supplementary Figure 3 Radiomics scores derived from five models for each patient. 0 (blue) represents N0/1 group, 1 (orange) represents N2 group.

Supplementary Figure 4 Calibration performance of five predictive models in two independent cohorts.
