# Supplementary figures and images for: Development and validation of machine learning-based MRI radiomics models for preoperative lymph node staging in T3 rectal cancer
Source: Front Oncol. 2025 Sep 8;15:1610892. doi: 10.3389/fonc.2025.1610892 (PMC12450691; doi:10.3389/fonc.2025.1610892)

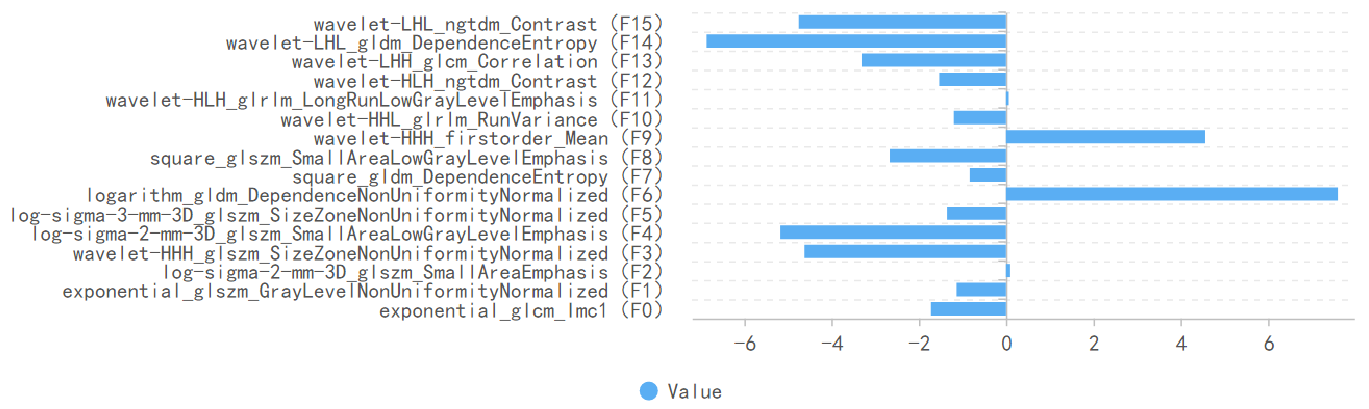

Supplement: Supplementary Figure 1 — Histogram of radiomics scores based on selected features. [file Image1.tif]

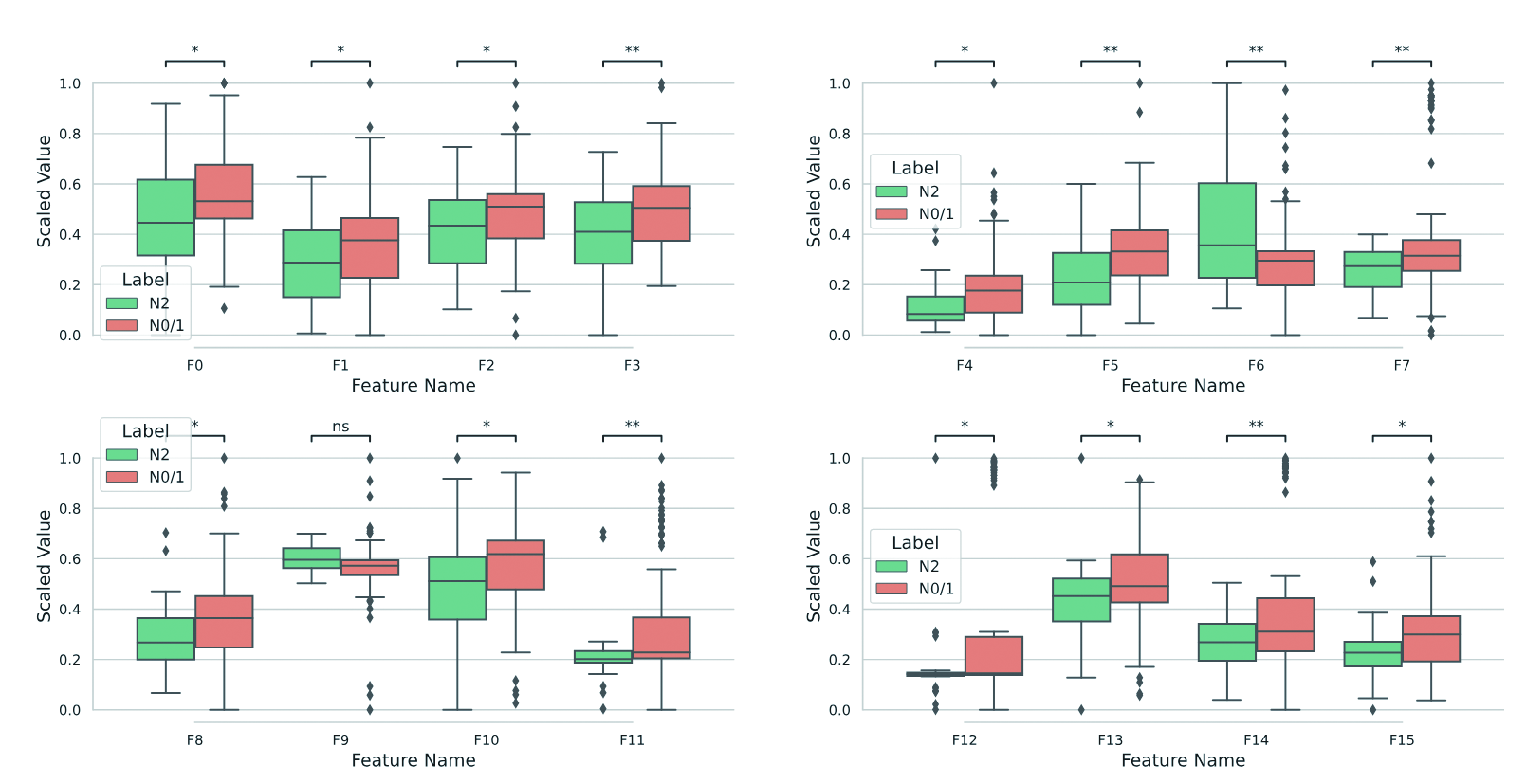

Supplement: Supplementary Figure 2 — Boxplots of 16 radiomics features in N0/1 and N2 groups. [file Image2.tif]

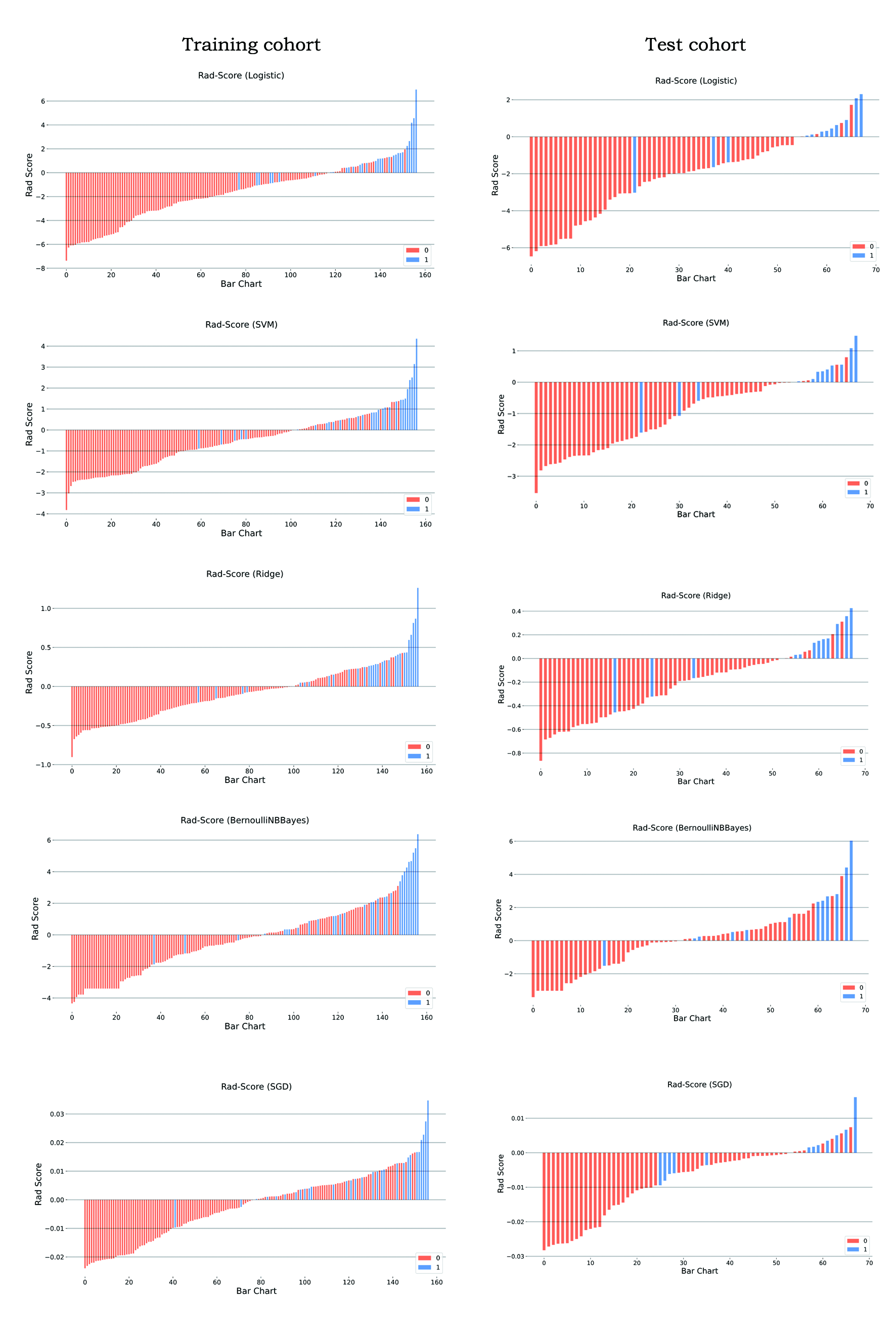

Supplement: Supplementary Figure 3 — Radiomics scores derived from five models for each patient. 0 (blue) represents N0/1 group, 1 (orange) represents N2 group. [file Image3.tif]

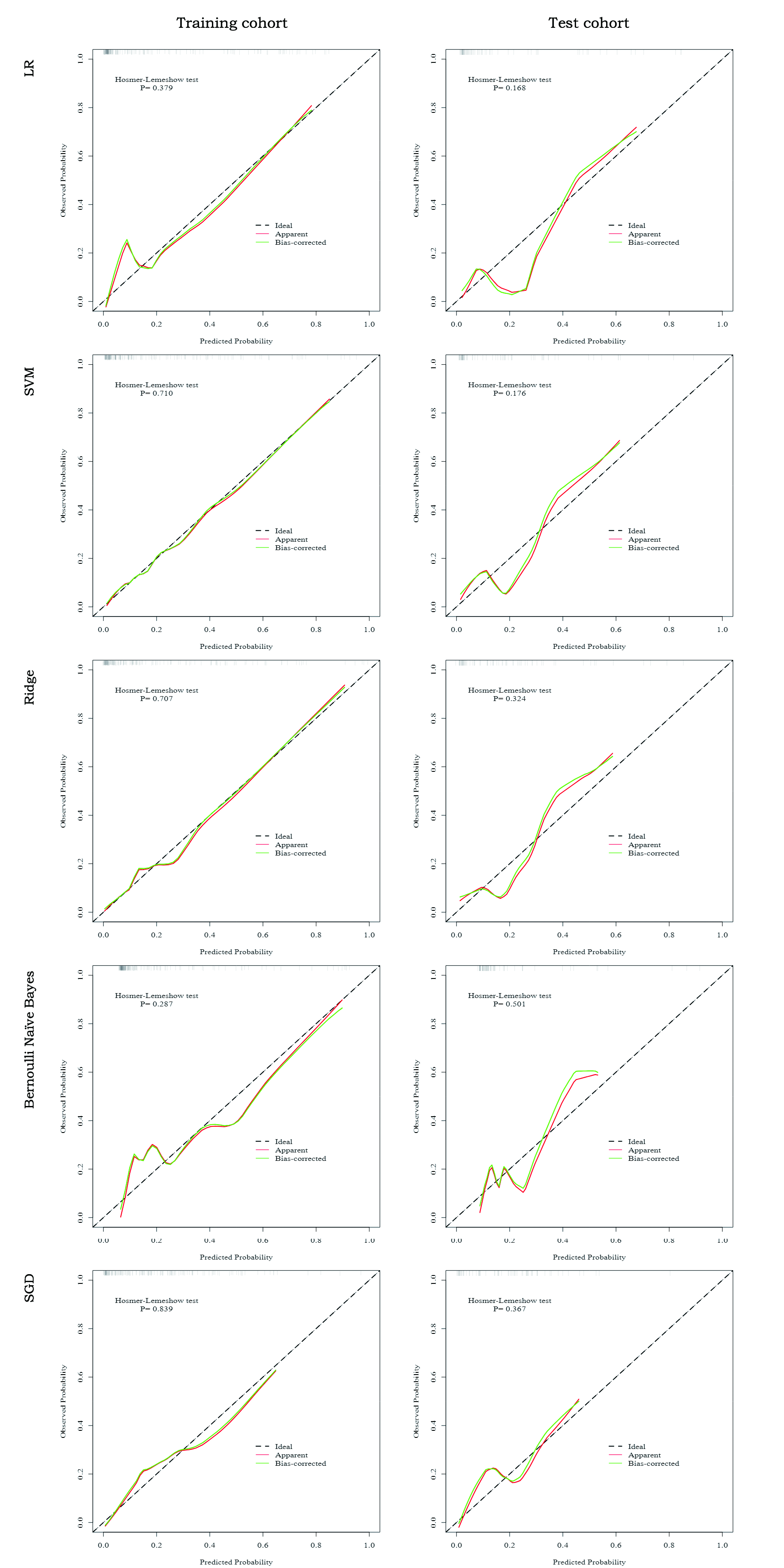

Supplement: Supplementary Figure 4 — Calibration performance of five predictive models in two independent cohorts. [file Image4.tif]
