# Supplementary material for: Development and validation of machine learning-based MRI radiomics models for preoperative lymph node staging in T3 rectal cancer
Source: Front Oncol. 2025 Sep 8;15:1610892. doi: 10.3389/fonc.2025.1610892 (PMC12450691; doi:10.3389/fonc.2025.1610892)
Supplement: Supplementary file 5 [file Table1.doc]

**Supplementary Table 1.** DeLong’s test results for ROC curves in different cohorts

|  | Variables | LR | SVM | Bernoulli Naïve Bayes | Ridge | SGD |
| --- | --- | --- | --- | --- | --- | --- |
| Training | LR | 1.000 | 0.094 | 0.035 | 0.365 | 0.005 |
|  | SVM | 0.094 | 1.000 | 0.171 | 0.237 | 0.020 |
|  | Bernoulli Naïve Bayes | 0.035 | 0.171 | 1.000 | 0.096 | 0.443 |
|  | Ridge | 0.365 | 0.237 | 0.096 | 1.000 | 0.009 |
|  | SGD | 0.005 | 0.020 | 0.443 | 0.009 | 1.000 |
| Test | LR | 1.000 | 0.344 | 0.037 | 0.080 | 0.050 |
|  | SVM | 0.344 | 1.000 | 0.078 | 0.083 | 0.066 |
|  | Bernoulli Naïve Bayes | 0.037 | 0.078 | 1.000 | 0.229 | 0.793 |
|  | Ridge | 0.080 | 0.083 | 0.229 | 1.000 | 0.203 |
|  | SGD | 0.050 | 0.066 | 0.793 | 0.203 | 1.000 |

^*^Difference was significant at the given level.
